# Intermediate scattering function of an anisotropic active Brownian particle

**DOI:** 10.1038/srep36702

**Published:** 2016-11-10

**Authors:** Christina Kurzthaler, Sebastian Leitmann, Thomas Franosch

**Affiliations:** 1 Institut für Theoretische Physik, Universität Innsbruck, Technikerstraβe 21A, A-6020 Innsbruck, Austria

## Abstract

Various challenges are faced when animalcules such as bacteria, protozoa, algae, or sperms move autonomously in aqueous media at low Reynolds number. These active agents are subject to strong stochastic fluctuations, that compete with the directed motion. So far most studies consider the lowest order moments of the displacements only, while more general spatio-temporal information on the stochastic motion is provided in scattering experiments. Here we derive analytically exact expressions for the directly measurable intermediate scattering function for a mesoscopic model of a single, anisotropic active Brownian particle in three dimensions. The mean-square displacement and the non-Gaussian parameter of the stochastic process are obtained as derivatives of the intermediate scattering function. These display different temporal regimes dominated by effective diffusion and directed motion due to the interplay of translational and rotational diffusion which is rationalized within the theory. The most prominent feature of the intermediate scattering function is an oscillatory behavior at intermediate wavenumbers reflecting the persistent swimming motion, whereas at small length scales bare translational and at large length scales an enhanced effective diffusion emerges. We anticipate that our characterization of the motion of active agents will serve as a reference for more realistic models and experimental observations.

Active particles are intrinsically out of equilibrium and exhibit peculiar dynamical behavior^1^,^2^,^3^,^4^,^5^ on the single as well as on the collective level. These active agents are ubiquitous in nature and include bacteria^6^,^7^,^8^,^9^, algae^10^, unicellular protozoa^11^,^12^,^13^ or spermatozoa^14^,^15^, that move due to a single or an array of flagella pushed by molecular motors. Only recently, artificial active particles have been synthesized and are self-propelled by either biomimetic motors^16^,^17^, or due to the response of their patterned surface to chemical or temperature gradients, thereby converting chemical energy into directed motion^18^,^19^,^20^,^21^,^22^. Furthermore, they also move in crowded media and their effective swimming speed is strongly determined by the viscoelasticity and geometrical constraints of the surroundings^23^,^24^.

To capture analytically the intricacies of the propulsion mechanisms, simple models for single swimmers have been conceived on different levels of coarse-graining. Microscopic theories for squirmers^25^,^26^, linked-bead swimmers^27^,^28^,^29^, self-thermophoresis^19^, and, self-diffusiophoresis^30^ of Janus particles have been elaborated and include the full hydrodynamic flow. On a larger scale, effective models for individual self-propelled particles ignoring hydrodynamics and the origin of the swimming motion are used to describe the stochastic motion and the dynamic behavior. There, the dynamics is modeled in terms of non-equilibrium Langevin equations^1^,^21^,^31^,^32^ such that the noise strength is an effective parameter unrelated to the temperature of the environment, in striking contrast to the fluctuation-dissipation theorem for equilibrium dynamics. In particular, these equations of motion serve as a suitable starting point for simulations^33^.

The complexity of the transport properties has often been quantified experimentally and in simulations in terms of low-order moments of the displacements^18^,^20^,^24^ and compared to theoretical models. For example, generically the mean-square displacement exhibits a regime resembling ballistic motion which directly reflects the persistent swimming. Only at longer times the motion becomes randomized and the mean-square displacement increases as anticipated from conventional diffusion. Higher moments can be derived^20^ in principle from the stochastic equations of motion, yet the calculations become more and more cumbersome with increasing order. However, these low-order moments provide only restricted information on the statistical properties of the random displacements as a function of time, in particular, they are to a large extend insensitive to the shape of the probability distribution.

More general spatiotemporal information is encoded in the intermediate scattering function *F*(*k*, *t*), which resolves the motion of the particle at lag time *t* on a length scale 2*π*/*k*, and is directly measurable in scattering experiments^34^ such as dynamic light scattering. The same quantity can be obtained by advanced image analysis within the recently developed differential dynamic microscopy (DDM)^35^,^36^, which provides direct access to the relevant length scales of active particles. Of course, single-particle tracking also collects the full statistical information and the intermediate scattering function can be obtained from this information, yet often the temporal resolution is not high enough to monitor the dynamics on small length scales. Last, the intermediate scattering function can also be viewed as the characteristic function^37^ of the random displacements, which is equivalent to the full probability distribution. In particular, the moments of the displacements are encoded as derivatives with respect to the wavenumber. Theoretical approaches to the intermediate scattering function for active particles are rare^38^ and no exact solutions appear to be available.

## Dynamics of an Active Brownian Particle

### Model

**Figure 1:**
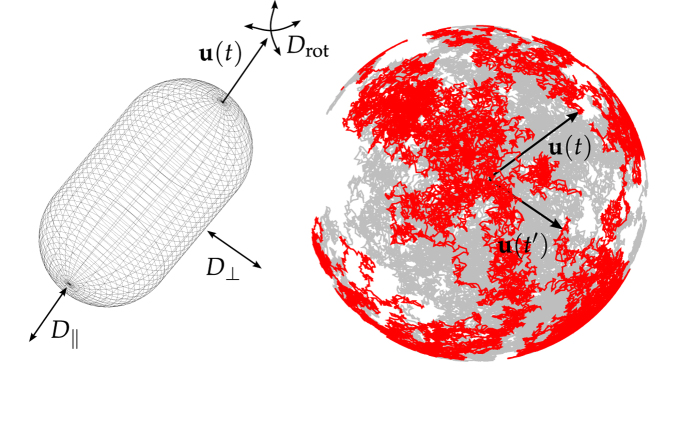
Model set up. Left: Anisotropic particle with orientation **u**(*t*) and translational *D*_||_, *D*_⊥_ and rotational *D*_rot_ diffusion coefficients. Right: Diffusion of the orientation **u**(*t*) on the unit sphere.

We assume the active Brownian particle to move at constant velocity *v* along its instantaneous orientation **u**(*t*) subject to random fluctuations determined by the rotational diffusion coefficient *D*_rot_. This diffusion process can geometrically be regarded as the diffusion of the orientation **u**(*t*) on the unit sphere, as in Fig. 1. In addition, the motion of the anisotropic active particle is characterized by axisymmetric translational diffusion measured in terms of the short-time diffusion coefficients parallel (*D*_||_) and perpendicular (*D*_⊥_) to the anisotropic particle, Fig. 1. Hence, for a three-dimensional swimmer the dynamics are described by the Langevin equations in It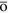 form for the position **r**(*t*) and the orientation **u**(*t*)


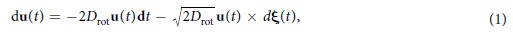



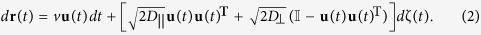


Here the diffusion coefficients *D*_||_ and *D*_⊥_ for the motion along and perpendicular to the axis of the swimmer encode the translation-rotation coupling. The random fluctuations are modeled in terms of independent white-noise processes, **ξ**(*t*) and **ζ**(*t*) with zero mean and covariance 〈*ξ*_*i*_(*t*)*ξ*_*j*_(*t*′)〉 = 〈*ζ*_*i*_(*t*)*ζ*_*j*_(*t*′)〉 = *δ*_*ij*_*δ*(*t* − *t*′) for *i*, *j* = 1, 2, 3. The drift term in Eq. (1) ensures that the normalization condition remains fulfilled, d[**u**(*t*)^2^]/d*t* = 0. Let us emphasize that if the Stratonovich interpretation is used, the drift term in the equation for the orientation needs to be dropped.

The model contains two dimensionless parameters, first the translational anisotropy Δ*D* = *D*_||_ − *D*_⊥_ relative to the mean diffusion coefficient 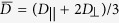. For passive rod-like particles in the limit of very large aspect ratio hydrodynamics suggests *D*_||_ = 2*D*_⊥_^39^, such that 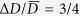. Here we consider *D*_||_ and *D*_⊥_ as effective parameters quantifying the noise only, and the anisotropy can take arbitrary values in 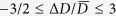. Next, the problem displays a characterstic length, 
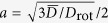
, which corresponds to the geometric radius of a spherical particle in the case of equilibrium diffusion coefficients *D*_rot_ = *k*_B_*T*/8*πηa*^3^ and 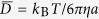. Then the second dimensionless parameter is the Péclet number 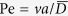 measuring the relative importance of the active motion with respect to diffusion.

### Analytic solution

From the stochastic differential equations one derives the Fokker-Planck equation^37^,^40^ for the time evolution of the probability density 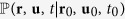 to find the swimmer at position **r**, with orientation **u** at time *t* given that it has been at some position **r**_0_ with initial orientation **u**_0_ at an earlier time *t*_0_. Since the stochastic process is translationally invariant in time and space, only displacements Δ**r** = **r** − **r**_0_ and lag times *t* (with *t*_0_ = 0) have to be considered, 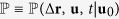. Then the Fokker-Planck equation assumes the form


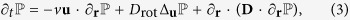


subject to the initial condition 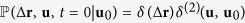, where the delta function on the surface of the sphere *δ*^(2)^(⋅, ⋅) enforces both orientations to coincide. Here, ∂_**r**_ denotes the spatial gradient, Δ_**u**_ the angular part of the Laplacian, reflecting the orientational diffusion, and 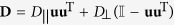. The first term on the right describes the active motion, in addition to the standard Smoluchowski-Perrin equation^39^ for the diffusion of an anisotropic particle. The Fokker-Planck equation for 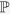 simplifies upon a spatial Fourier transform


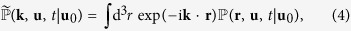


which solves the equation of motion


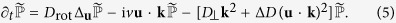


The quantity of interest in scattering experiments^34^ is the intermediate scattering function (ISF)


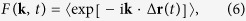


which is obtained by marginalizing over all final orientations **u** and averaging over all initial orientations **u**_0_,


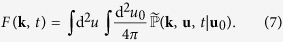


The ISF can also be interpreted as the characteristic function^37^ of the random displacement variable Δ**r**(*t*). In particular, the moments are obtained by taking derivatives with respect to the wave vector **k**. Since after averaging the motion is isotropic, the ISF *F*(*k*, *t*) ≡ *F*(**k**, *t*) depends only on the magnitude of the wave vector *k* = |**k**|. Averaging over the directions of **k** yields the equivalent representation


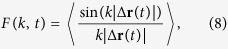


and the expansion of the ISF for small wavenumbers


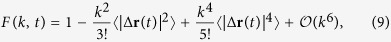


allows one to recover the mean-square displacement 〈|Δ**r**(*t*)|^2^〉 and the mean-quartic displacement 〈|Δ**r**(*t*)|^4^〉 by comparing the corresponding terms in the small-wavenumber expansion. More generally, even moments can be obtained numerically by taking derivatives of the ISF with respect to the squared wavenumber,


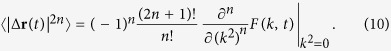


The equation of motion, Eq. (5), is reminiscent of a Schrödinger equation on the unit sphere and can be solved by separation of variables. We parametrize the orientation **u** = (sin *ϑ* cos *φ*, sin *ϑ* sin *φ*, cos *ϑ*)^*T*^ in terms of its polar angles, and similarly for **u**_0_. Then the solution is a superposition of appropriate eigenfunctions


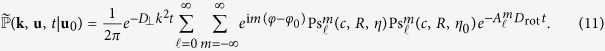


Here we abbreviated *η* = cos *ϑ*, *η*_0_ = cos *ϑ*_0_, and 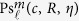 are the generalized spheroidal wave functions of order *m* and degree 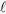
41,42,43. They solve the corresponding eigenvalue problem


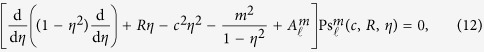


with eigenvalue 
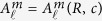
 and we identify the dimensionless parameters *R* = −i*kv*/*D*_rot_ and *c*^2^ = Δ*Dk*^2^/*D*_rot_. Hence, at fixed wavenumber *k*, *R* parametrizes the importance of active motion with respect to orientational diffusion, whereas *c* measures the coupling of the translational and orientational diffusion. In particular, the ratio 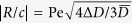 is wavenumber-independent.

Integrating Eq. (11) over the polar angles, only 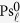 contributes and we obtain


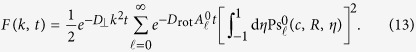


The explicit expression Eq. (13) for the intermediate scattering function *F*(*k*, *t*) in terms of the generalized spheroidal wave functions is one of the principal results of this work.

### Exact low moments

The low-order moments can be obtained upon expanding the ISF for small wave numbers (Eq. (13)) such that the moments can be identified with Eq. (9). Here we illustrate the derivation only for the mean-square displacement.

For *R* = 0 and *c*^2^ = 0 the spheroidal wave functions reduce to the Legendre polynomials, 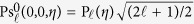 with eigenvalues 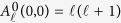. For small dimensionless parameters *R*, *c* the Legendre polynomials are deformed analytically, to order 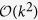, as required for the mean-square displacement, Eq. (9), the 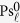 acquire contributions 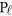, 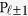, and, 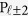, concomitantly the eigenvalues 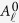 shift. The explicit expressions are lengthy and deferred to the methods section. The integral in Eq. (13) can then be performed using the orthogonality of the Legendre polynomials and one concludes that only terms 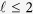 need to be taken into account to order 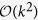. Yet, inspection of Eq. (19) of the methods section shows that integration of 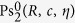 yields terms of order 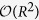 and 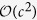 and after squaring in Eq. (13) of only order 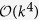. Hence, the contributing eigenfunctions for the mean-square displacement evaluate to


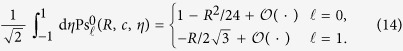


and the corresponding eigenvalues read


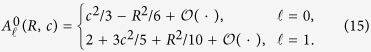


Collecting results for the ISF *F*(*k*, *t*) to order 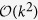 and comparing with Eq. (9), yields for the mean-square displacement


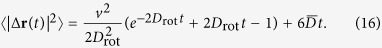


This expression generalizes the earlier result for the case of an isotropic active agent^31^,^38^ and anisotropic passive particle^39^,^44^. It also recovers the mean-square displacement of a freely rotating ellipsoidal particle^45^ obtained directly from the Langevin equations. Alternatively 〈|Δ**r**(*t*)|^2^〉 can be calculated by time-dependent perturbation theory from Eq. (5) up to second order.

The first contribution to the mean-square displacement in Eq. (16) reflects the active motion, which displays directed motion *v*^2^*t*^2^ for times 
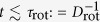
 where the particle does not change its direction significantly. During this time the particle covers a typical distance *L* = *v*/*D*_rot_, which we refer to as the persistence length. In contrast at times 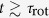 the active contribution increases linearly *v*^2^*t*/6*D*_rot_ where the orientational degree of freedom is relaxed. The second contribution is merely the isotropically averaged translational motion. Interestingly at the level of the mean-square displacement there is no coupling between the translational diffusion and the active motion induced by the orientational diffusion.

**Figure 2:**
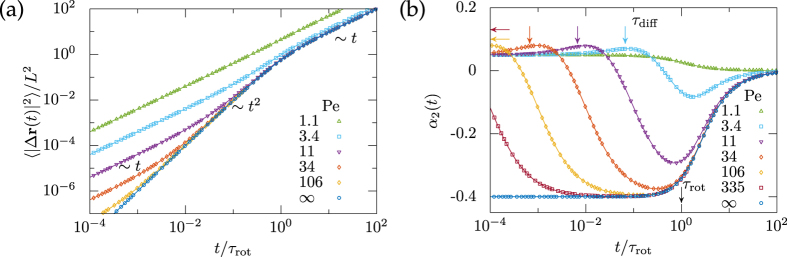
Exact low-order moments of a single self-propelled particle subject to translational Brownian motion with hydrodynamic anisotropy 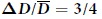. (**a**) Mean-square displacement 〈|Δ**r**(*t*)|^2^〉/*L*^2^ in units of the persistence length *L* = *v*/*D*_rot_, and, (**b**) non-Gaussian parameter *α*_2_(*t*) for different Péclet numbers, 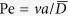. Simulation and theory results are shown using symbols and lines, respectively.

From the mean-square displacement we identify three temporal windows, Fig. 2(a). For short times 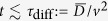 it increases linearly by the translational diffusion only, while at longer times the persistent swimming motion dominates. At even longer times 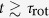 the mean-square displacement increases again linearly with an effective diffusion coefficient 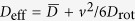, equivalently the enhancement is 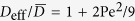. The crossover from persistent motion to effective diffusion occurs at length scale 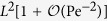. The window of persistent motion is set by the ratio of the two crossover times *τ*_rot_/*τ*_diff_ = 4Pe^2^/3 and opens upon increasing the Péclet number.

Extending the expansion of the intermediate scattering function up to fourth order in the wavenumber *k* is tedious and the result is lengthy,


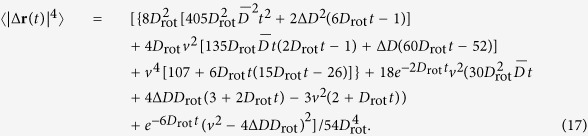


In contrast to the mean-square displacement, the mean-quartic displacement depends explicitly on the translational anisotropy Δ*D* such that the rotational-translational coupling becomes important. We shall see below that depending on Δ*D* the dynamics becomes qualitatively different.

Rather than the mean-quartic displacement, we focus on the non-Gaussian parameter^46^


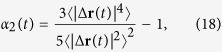


which is a sensitive indicator on how far the process deviates from diffusion, see Fig. 2(b).

For long times 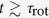 the non-Gaussian parameter approaches zero 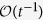 for all Péclet numbers as anticipated by the central limit theorem. Interestingly, for the limiting case of a self-propelled particle without any translational diffusion, Pe = ∞, one infers *α*_2_(*t* → 0) = −2/5, which reflects the persistent swimming motion at short-times. In contrast, for non-vanishing translational diffusion, Pe < ∞, the non-Gaussian parameter approaches a constant 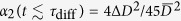 for short-times, as anticipated for anisotropic translational diffusion. In particular, for *D*_||_ = 2*D*_⊥_ it assumes the value 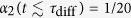, whereas it vanishes for isotropic diffusion. For large Péclet number there is an extended intermediate temporal regime, where the non-Gaussian parameter is close to the one for infinite Péclet number, thereby a prominent minimum emerges. Here the negative non-Gaussian parameter can be traced back to the directed swimming motion, which dominates the translational diffusion of the active agent at these intermediate times. Thus, for decreasing *τ*_diff_ the intermediate negative plateau of directed swimming motion in the non-Gaussian parameter is observed for longer times, see Fig. 2(b).

For the parameters shown in Fig. 2(b) an additional maximum occurs at shorter times. One can work out analytically from the initial slope of *α*_2_(*t*) that this happens only for positive anisotropies Δ*D* > 0 and Péclet numbers 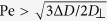. Conversely, we conclude that a maximum in the non-Gaussian parameter is a genuine fingerprint of active motion.

### Intermediate scattering function

**Figure 3:**
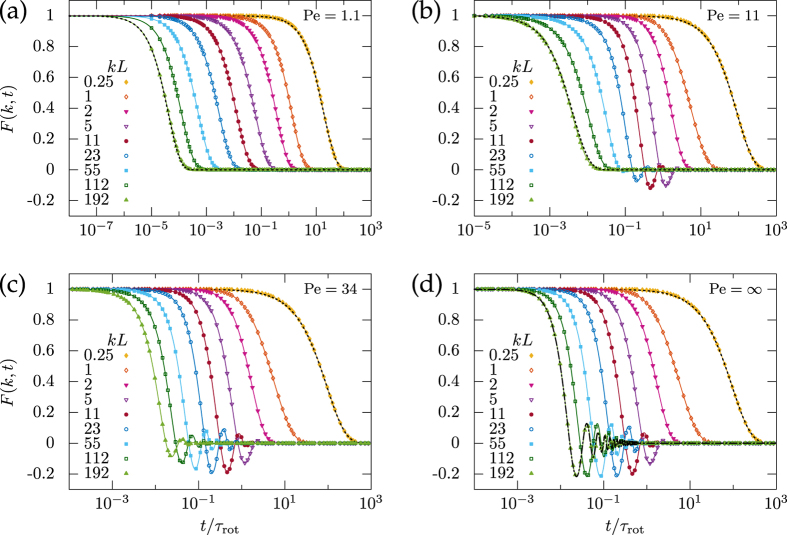
Intermediate scattering function *F*(*k*, *t*) of an active Brownian particle subject to translational diffusion (here 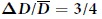) for the full range of wavenumbers *k* measured in terms of the persistence length *L* = *v*/*D*_rot_. The dashed line represents relaxing exponentials exp(−*D*_eff_*k*^2^*t*) and 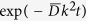 for small and large wavenumbers, respectively. The dashed-dotted line in (d) indicates the sinc function sin(*kvt*)/*kvt*.

We have evaluated numerically the series for the intermediate scattering function in Eq. (13) for arbitrary times and wavenumbers and compare the results to stochastic simulations, see Fig. 3. The natural scale for the wavenumbers *k* is set by the persistence length *L*, and our data cover the small length scales resolving the persistent swimming motion as well as large length scales where the particle undergoes a random walk. Indeed for small wavenumbers the ISF are well approximated by an effective diffusion, exp(−*D*_eff_*k*^2^*t*) with the effective diffusion coefficient obtained from the long-time behavior of the mean-square displacement. Increasing the wavenumber the qualitative behavior depends on the Péclet number.

For small Péclet number (see Fig. 3(a)) the ISF decreases monotonically for all wave numbers, in particular, the large wavenumbers approach again an exponential 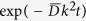 characterized by the mean translational diffusion coefficient 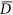. This behavior is consistent with the linear increase of the mean-square displacement, Fig. 2(a), for small Péclet numbers. For intermediate wavenumbers (Fig. 3(b,c)) the shape of the ISF is no longer a pure exponential since the translation-rotation coupling becomes relevant at time scales 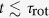.

For Péclet numbers, 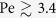, the ISF displays damped oscillations for wavenumbers that start to resolve the motion on the scale of the persistence length. At length scales 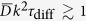 short-time diffusion takes over again, see Fig. 3(b). Inserting the definition of *τ*_diff_, one infers that this regime corresponds to length scales 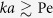 where the swimmer moves only a fraction of its size *a*. In particular, for high Péclet numbers 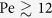 the short-time diffusion is no longer resolved for the wavenumbers shown in Fig. 3(c). For infinite Péclet number, the translational diffusion is negligible and the ISF oscillates for wavenumbers resolving the persistence length, Fig. 3(d).

The physics of these oscillations can be rationalized easily by inspecting the general expression of the ISF, Eq. (8). For wavenumbers such that the rotational and translational diffusion can be ignored, the trajectories can be approximated by purely persistent motion |Δ**r**(*t*)| = *vt* and there the ISF follows *F*(*k*, *t*) = sin(*vkt*)/*vkt*, as has been discussed already in Ref. 34. For infinite Péclet number the sinc function serves as a good approximation for wavenumbers 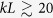.

**Figure 4:**
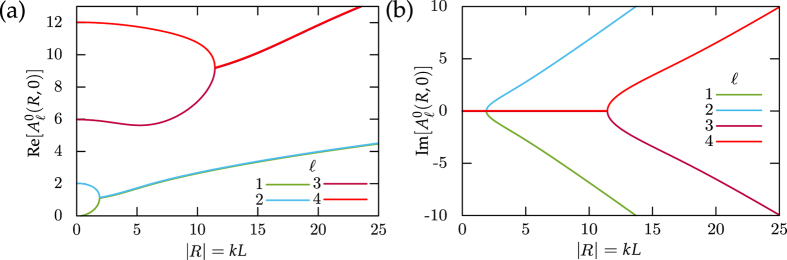
Real (**a**) and imaginary (**b**) part of the eigenvalues 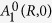 to 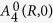 for vanishing translational diffusion (Pe = ∞).

It is also interesting to ask how the oscillations emerge mathematically from the general solution in terms of eigenfunctions, Eq. (13). Naively, one expects that the ISF is a sum of relaxing exponentials only, in particular, they should decay monotonically. Yet, the operator in Eq. (12) for the eigenvalue problem is non-Hermitian, since *R* = −i*kL* is not real, such that the eigenvalues can become complex. Indeed one can show (see section Methods, Fig. 4), for example Pe = ∞, that at |*R*| = 1.9 the two lowest real eigenvalues merge and bifurcate to a pair of complex conjugates. Further bifurcations for larger eigenvalues occur at even larger |*R*|. For large Péclet numbers the scenario is qualitatively similar, whereas for small Pe the eigenvalues remain real and no oscillations in the ISF emerge. Since the eigenvalues depend non-analytically on |*R*| = *kL*, there is a finite radius of convergence for the expansion of the ISF in powers of *k* set by the first bifurcation point. In particular, the oscillations cannot be obtained by extending the series expansion, Eq. (9), in terms of the moments to arbitrary order.

## Summary and Conclusion

We have determined exact analytic expressions for the intermediate scattering function (ISF) of an anisotropic active Brownian particle in terms of an expansion of eigenfunctions. The solution is validated and exemplified by stochastic simulations. Interestingly, the ISF displays a regime with oscillatory behavior in striking contrast to passive motion in equilibrium systems. These oscillations are rationalized in terms of bifurcations of the eigenvalue problem and reflect the directed swimming motion of the active particles. In addition to the mean-square displacement, we have analyzed the non-Gaussian parameter and identified a characteristic maximum for positive anisotropies and large Péclet numbers.

The non-Gaussian parameter has been derived before for two-dimensional isotropic swimmers^31^,^38^ by a truncated mode expansion of the Fokker-Planck equation. Yet, for isotropic diffusion the non-Gaussian parameter remains negative for all times, in contrast to experimental observations^20^. The mode expansion also yields approximate expressions for the ISF which in principle also display oscillations in time for the two-dimensional case.

In differential dynamic microscopy experiments for dilute suspensions of *E. coli* bacteria in three dimensions an oscillatory behavior for the ISF has been observed and analyzed approximately in terms of pure persistent swimming motion^36^. Our results predict that these oscillations fade out for large as well as small wavenumbers which should in principle be also measurable in the set-up. The motility parameters then can be extracted from the measured ISF relying on different wavenumbers. The dynamics on small length scales is dominated by translational diffusion, at intermediate ones by the swimming motion, and finally at large length scales by the rotational diffusion.

Furthermore the spatio-temporal information obtained from the ISF allows to discriminate quantitatively the dynamics of different swimming behaviors, whereas the mean-square displacement of several models such as simple run-and-tumble motion^47^ is hardly distinguishable from that of an active Brownian particle.

The analytic solution for the active Brownian swimmer derived here should serve as a reference for more complex swimming behavior. For example, *E. coli* bacteria display a distribution of swimming velocities, which can be accounted for directly by post-averaging our results for the ISF. Similarly, the swimming velocity may fluctuate itself^1^ leading to a further smearing of the oscillations in the ISF. Furthermore, the rotational diffusion for bacteria should be complemented by a run-and-tumble motion^6^ as observed by particle tracking. Species-specific propulsion mechanisms, such as circular motion of the algae *Chlamydomonas reinhardtii*^36^, can be accounted for by introducing a torque in the Fokker-Planck equation. Our solution strategy can be adapted also to two-dimensional systems, for instance for the movement of Janus particles^20^ confined between two glass plates or for the circular motion of *E. coli* bacteria close to surfaces^8^.

## Methods

### Expansion of the eigenfunctions in powers of the wavenumber

The starting point of the expansion are the reference solutions 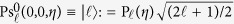 for 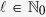 of the eigenvalue problem, Eq. (12), for parameters *R* = *c*^2^ = 0. By standard perturbation theory one derives to the desired order 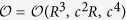



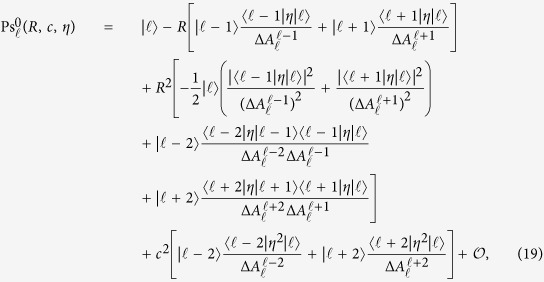


with corresponding eigenvalues


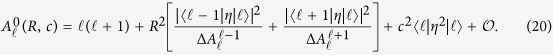


Here 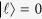 for 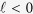, the difference of unperturbed eigenvalues is denoted by 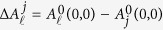, and the matrix elements of the perturbation


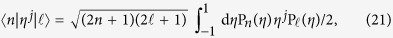


for *j* = 1, 2 can be evaluated using the properties of the Legendre polynomials.

### Numerical evaluation of the ISF

For the ISF we need the eigenvalues 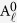 and the integrals over the eigenfunctions 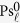, Eq. (13). We expand these in terms of the Legendre polynomials^41^ 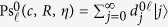. Then the integrals in Eq. (13) can be performed and the intermediate scattering function of the anisotropic active Brownian particle reads


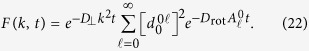


Inserting the expansion into Eq. (12) and projecting onto 〈*n*| leads to the matrix eigenvalue problem


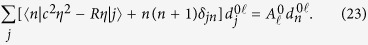


Since the matrix elements are non-vanishing for *j* = *n* − 2, ..., *n* + 2 only, it is in fact a band matrix with two diagonals on each side. Then the normalized eigenvectors 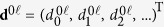 and eigenvalues 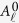 can be efficiently determined numerically. In practice we truncate the matrix in Eq. (23) to sufficiently high order such that the normalization at time *t* = 0 for the ISF, Eq. (22), is achieved. Since the generalized spheroidal wave equation is not Hermitian, the corresponding eigenvalues can become complex. In fact for Pe = ∞ (*c* = 0), the two lowest eigenvalues merge at |*R*| = *kL* = 1.9 and a bifurcation to two complex conjugates occurs, see Fig. 4. In contrast for small Pèclet number Pe = 1.1 the eigenvalues remain real for all wavenumbers.

## Additional Information

**How to cite this article**: Kurzthaler, C. *et al.* Intermediate scattering function of an anisotropic active Brownian particle. *Sci. Rep.* **6**, 36702; doi: 10.1038/srep36702 (2016).

**Publisher’s note:** Springer Nature remains neutral with regard to jurisdictional claims in published maps and institutional affiliations.
