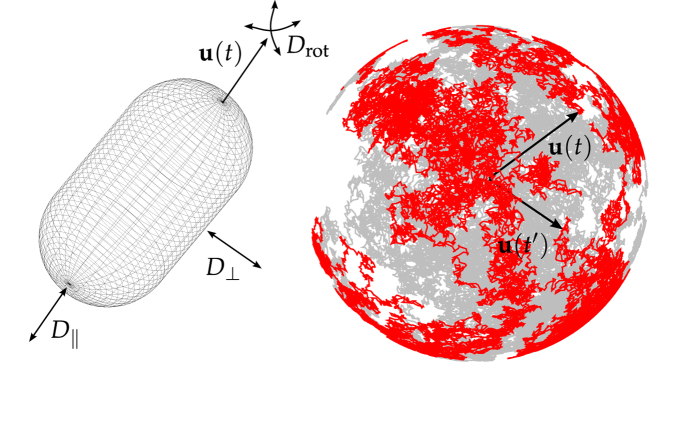# Erratum: Intermediate scattering function of an anisotropic active Brownian particle

**DOI:** 10.1038/srep39577

**Published:** 2017-01-04

**Authors:** Christina Kurzthaler, Sebastian Leitmann, Thomas Franosch

Scientific Reports 6: Article number: 3670210.1038/srep36702; published online: 11 10 2016; updated: 01 04 2017

The original version of this Article contained errors. The publication date of the Article, 10th November 2016, was incorrectly listed as 10th October 2016.

In addition, an incorrect version of Figure 1 was published where “Drot” was omitted.

There were typographical errors in formulas (1) and (2):


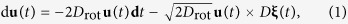



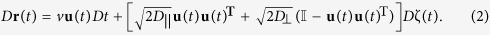


now read:


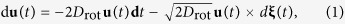



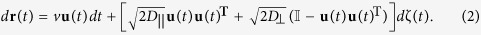


These errors have now been fixed in the HTML and PDF versions of this Article.

## Figures and Tables

**Figure 1:**